# A quasi-experimental study provides evidence that registered dietitian nutritionist care is aligned with the Academy of Nutrition and Dietetics evidence-based nutrition practice guidelines for type 1 and 2 diabetes

**DOI:** 10.3389/fnut.2022.969360

**Published:** 2022-09-12

**Authors:** Erin Lamers-Johnson, Kathryn Kelley, Kerri Lynn Knippen, Kimberly Feddersen, Damien M. Sánchez, J. Scott Parrott, Casey Colin, Constantina Papoutsakis, Elizabeth Yakes Jimenez

**Affiliations:** ^1^Academy of Nutrition and Dietetics, Chicago, IL, United States; ^2^Department of Public and Allied Health, Bowling Green State University, Bowling Green, OH, United States; ^3^Federal Bureau of Prisons, Rochester, MN, United States; ^4^Organization, Information, and Learning Sciences, University of New Mexico, Albuquerque, NM, United States; ^5^Department of Interdisciplinary Studies, Rutgers University, Blackwood, NJ, United States; ^6^Nutrition and Dietetics Department, University of North Florida, Jacksonville, FL, United States; ^7^Departments of Pediatrics and Internal Medicine and College of Population Health, University of New Mexico, Albuquerque, NM, United States

**Keywords:** medical nutrition therapy (MNT), type 2 diabetes, type 1 diabetes, nutrition care process (NCP), dietitians

## Abstract

**Background:**

One previous study examined implementation of evidence-based nutrition practice guidelines (EBNPG).

**Objectives:**

To describe alignment of registered dietitian nutritionists' (RDNs) documented nutrition care with the Academy of Nutrition and Dietetics' EBNPG for Type 1 and Type 2 diabetes and examine impact of a midpoint training on care alignment with the guideline.

**Methods:**

In this 2-year, quasi-experimental study, 19 RDNs providing outpatient medical nutrition therapy to adults with diabetes (*n* = 562) documented 787 initial and follow-up encounters. At study midpoint, RDNs received a guideline content training. A validated, automated tool was used to match standardized nutrition care process terminology (NCPT) in the documentation to NCPT expected to represent guideline implementation. A congruence score ranging from 0 (recommendation not identified) to 4 (recommendation fully implemented) was generated based on matching. Multilevel linear regression was used to examine pre-to-post training changes in congruence scores.

**Results:**

Most patients (~75%) had only one documented RDN encounter. At least one guideline recommendation was fully implemented in 67% of encounters. The recommendations “individualize macronutrient composition” and “education on glucose monitoring” (partially or fully implemented in 85 and 79% of encounters, respectively) were most frequently implemented. The mean encounter congruence scores were not different from pre-to-post guideline training (*n* = 19 RDNs, 519 encounters pre-training; *n* = 14 RDNs, 204 encounters post-training; β = −0.06, SE = 0.04; 95% CI: −0.14, 0.03).

**Conclusions:**

Most RDN encounters had documented evidence that at least one recommendation from the EBNPG was implemented. The most frequently implemented recommendations were related to improving glycemic control. A midpoint guideline training had no impact on alignment of care with the guideline.

## Introduction

Diabetes mellitus is a significant public health concern, with over 10% of the U.S. population diagnosed with type 1 or type 2 diabetes (1). The American Diabetes Association recommends that diabetes care adhere to evidence-based guidelines (2). Medical nutrition therapy (MNT) provided by a registered dietitian nutritionist (RDN) can help people with diabetes improve glycemic control, prevent and treat cardiovascular disease, optimize medication use, manage body weight, and improve quality of life (3–6). To assist RDNs, the Academy of Nutrition and Dietetics (Academy) Evidence Analysis Center developed the Diabetes Type 1 and 2 Evidence-Based Nutrition Practice Guideline (Diabetes EBNPG) (3, 7, 8). The guideline contains evidence-based recommendations organized by the four steps of the Nutrition Care Process (NCP) Model (9).

Previously, a single pilot study compared RDN care to the Diabetes Prevention EBNPG recommendations before and after a midpoint training on the guideline. The authors found a small (4%) improvement in the percentage of care that reflected guideline recommendations from pre-to-post training (10). There has been more extensive study of guideline implementation among other health professionals. A systematic meta-review of systematic reviews examining factors that influence implementation of clinical guidelines found that factors at multiple levels (i.e., guideline, patient, provider, clinical environment) have important influence on guideline implementation (11). Generating more real-world evidence on EBNPG implementation by RDNs can help justify the substantial resources invested in creating quality EBNPGs and guide EBNPG development and implementation strategies in the future (7).

To examine implementation of the Diabetes EBNPG, we conducted a study to evaluate alignment of RDNs' documented nutrition care with the Academy's evidence-based nutrition practice guideline for Type 1 and Type 2 diabetes. We also investigated the impact of a midpoint guideline training on alignment with the guideline. We hypothesized that a targeted midpoint training would improve the alignment of RDN care with the guideline.

## Methods

### Study design

This quasi-experimental study used a controlled pre-post design and was conducted from May 2017 to June 2019 (see Supplementary material for study timeline) (12, 13). The focus was on measuring implementation of the guidelines under real world conditions (13). Outpatient RDNs providing MNT to adult patients with type 1 or 2 diabetes documented initial and follow-up nutrition care for a randomly selected subset of their patients for 11–14 months and then completed training on Diabetes EBNPG content. RDNs then documented initial and follow-up care for a new randomly selected subset of their patients for 11 months. Documentation detailing assessment, diagnosis, intervention, and monitoring/evaluation activities for each encounter was entered into the Academy of Nutrition and Dietetics Health Informatics Infrastructure (ANDHII) (14). ANDHII is a web-based platform designed to collect de-identified nutrition care data in the NCP framework using nutrition care process terminology (NCPT) (9, 14, 15).

### Ethical approval

The American Academy of Family Physicians Institutional Review Board determined the project was not research involving human subjects based on Office for Human Research Protections Guidance on Research Involving Coded Private Information or Specimens (#17-287) (16). Based on this guidance, the identities of individuals whose data are documented are protected from disclosure to the investigators, and clinical data are not obtained through research interventions. RDNs documented routine care, meaning that the care provided and documentation recorded were not standardized across sites, beyond use of the NCPT to document.

### RDN recruitment, training, and surveys

Registered dietitian nutritionists were recruited *via* an open call to members of the Academy's Diabetes Dietetic Practice Group and Nutrition Research Network. RDNs were eligible to enter the study if they regularly provided outpatient nutrition care to adult patients with diabetes and were able to travel to one of four in-person trainings. Between May and July 2017, 33 RDNs attended 1 of the 5-h in-person trainings that focused on navigating and using the Evidence Analysis Library, electronic NCPT, and ANDHII platforms. Twenty-four RDNs obtained institutional approval to participate, and 19 RDNs began documenting nutrition care from June to September 2017. There was some variability in when RDNs began documenting care because the time required to receive institutional approval varied across sites. Fifteen RDNs provided information on their education level, training, and facility characteristics.

At the study midpoint (July 2018), RDNs were invited to complete virtual trainings (2.5 continuing professional education units) on the 13 imperative intervention recommendations of the Diabetes EBNPG and continuous quality improvement methods (8). The training was completed by 16 RDNs.

Knowledge of the Diabetes EBNPG was assessed before and after the training using a 12-question test on the training content. Each question was worth one point for a correct answer. Fifteen RDNs completed both pre- and post-training knowledge tests.

### ANDHII documentation

Adult patients were eligible to have their care documented into ANDHII if they were attending their first outpatient visit with the RDN for diabetes MNT and were referred with a primary diagnosis of diabetes. Patients were excluded if they did not have a confirmed diagnosis of diabetes or had previously seen the participating RDN for diabetes MNT. To randomly select patients who had their care documented, the RDNs used a random number generator to produce two random numbers within a range from 1 (first new patient assessment of the month) to the total number of new patient assessments in the month (17). The generated random numbers were used to determine which two patients on the list of new assessments had their care included in the registry.

Registered dietitian nutritionists documented nutrition care into ANDHII before and after the midpoint training. For both the period before and after the EBNPG training, RDNs were asked to document care for at least two new patients per month, and then record any follow-up nutrition encounters for those patients until July 2018 and June 2019, respectively. They were reminded to document monthly *via* a study communication portal. For 42 patients from the pre-training period, the RDNs erroneously continued to enter follow-up encounters into the post-training period; these entries (*n* = 64 encounters) were included in analyses that spanned the whole study period but were removed from analyses comparing the pre- and post-training periods. Three documented encounters were excluded from all analyses, as they were completed directly after the study training by one RDN who then stopped participating. RDNs who documented at least one encounter per month were eligible to win a $25 gift card.

### Assessment of alignment of care with the diabetes EBNPG

The methods used to assess alignment of RDN care with the Diabetes EBNPG have been described in detail elsewhere (10, 18). In brief, recommendations from the Diabetes EBNPG were transformed *a priori* from guiding statements into Expected Care Plans (ECPs) by Academy Nutrition Research Network staff and an advisory group that included RDNs experienced in diabetes care. An ECP is a defined set of NCPT that are expected to be present in documentation when an RDN is implementing care that is aligned with a specific guideline recommendation and when continuous clinical judgment has occurred during application (10, 19, 20).

The Diabetes ECP Analyzer, a validated natural language processing tool, was used to compare RDNs' documented care to the pre-established ECPs. The Analyzer was built in Excel 2019 using adapted (21), custom Visual Basic for Applications code. The Analyzer counted the number of matching NCPT between RDNs' documentation for an encounter and the ECPs for the 13 imperative intervention recommendations from the Diabetes EBNPG (18). A congruence score of 0–4 points was assigned to each encounter based on the pattern of matching terms (Table 1) and was then classified on a five-point scale: “[Diabetes EBNPG] recommendation not identified” (0 points), “recommendation identified, but not implemented” (1 point), “recommendation partially implemented” (2–3 points), or “recommendation fully implemented” (4 points). An algorithm was used to account for encounters that produced matching terms in both the evidence component and the monitoring/evaluation component, but not in the diagnosis or intervention components; the score was corrected to 1 point, or “identified, but not implemented,” because the evidence was not addressed in the diagnosis or intervention. Certain partially implemented recommendations were classified as “deferred” (no matching ECP terms in the evidence component) or “interrupted” (matching ECP terms in the evidence component but not in a subsequent component [diagnosis or intervention]) (14). An example of an interrupted partially implemented recommendation could include a diagnosis of “excessive carbohydrate intake” and a documented intervention of “increase physical activity.” In this example, there is no logical connection between the diagnosis and the intervention. In other words, an intervention would have to be nutritional in nature to address a nutrient intake problem like “excessive carbohydrate intake.”

**Table 1:** Recommendation-level match criteria for the expected care plans (ECP) in the diabetes ECP analyzer.

**Evidence**	**Diagnosis**	**Intervention**	**Monitoring & evaluation**	**Score**	**Classification^a^**
				0	Not identified
Match			Match	1 point^b^	Identified, not implemented [Incomplete]
Match		Match		2 points	Partially implemented [Incomplete]
Match	Match			2 points	Partially implemented [Incomplete]
	Match	Match		2 points	Partially implemented [Deferred]
Match		Match	Match^a^	2 points	Partially implemented [Interrupted]
Match	Match		Match^a^	2 points	Partially implemented [Interrupted]
	Match	Match	Match	3 points	Partially implemented [Deferred]
Match	Match	Match		3 points	Partially implemented [Incomplete]
Match	Match	Match	Match	4 points	Fully implemented

a Partially implemented recommendations with no matching ECP terms in the evidence component were considered “deferred.” Partially implemented recommendations with a matching ECP term in the evidence component but no matching ECP terms in a subsequent component (diagnosis or intervention) were considered “interrupted” (14).

b An algorithm was used to account for situations when an encounter produced matching terms in the evidence component and monitoring and evaluation component, but not in the diagnosis or intervention components. Though there were technically two matches in the encounter, the score was corrected to be a 1—“identified, but not implemented” because the evidence was not addressed in the diagnosis or intervention.

Overall guideline alignment for an encounter was assessed by determining if *at least* one of the 13 recommendations was partially implemented (congruence score of 2–3 points) or fully implemented (congruence score of 4 points) in the encounter. For encounters with at least one partially or fully implemented recommendation, an average congruence score (i.e., level of implementation across all identified recommendations in the encounter) was calculated. RDN care documented in an encounter was *not* considered to be congruent with the guideline if none of the 13 recommendations were partially or fully implemented (i.e., congruence score of 0–1 points for all 13 recommendations).

In addition, RDN documentation of the major outcome measures described in the Diabetes EBNPG was cataloged. These measures include: glycemia (HbA1c or glucose), medication use (insulin or other glucose-lowering medications), cardiovascular disease risk factors (lipids or blood pressure), quality of life, and weight management (body weight, BMI, or waist circumference).

### Statistical analysis

Statistical analyses were conducted using Stata SE 16.0 and SAS Version 9.4 (22, 23). Statistical significance was interpreted as values of *p* ≤ 0.05. Data are presented descriptively as means ± standard deviations (SD), medians and interquartile ranges (IQR), number of observations and respective percentages, or with graphs. Change in knowledge from pre-to-post training was examined using a Wilcoxon matched-pairs signed-rank test. Changes in congruence scores from pre-to-post training were evaluated using a multilevel mixed effects linear regression model including encounter level congruence score as the dependent variable, documentation period (pre- or post-training) as a covariate, and random effects of RDN and patient.

## Results

### RDN characteristics and documentation of care

The majority (80%) of RDNs (*n* = 15) worked in outpatient departments of hospitals or medical centers. They had an average of 12.9 ± 9.9 years of experience, and approximately one-fourth held an advanced degree.

In this 2-year study, 19 RDNs documented 787 encounters for 562 patients. All 19 RDNs documented during the pre-training period, and of these, 14 RDNs documented during the post-training period. About one-fourth of patients (*n* = 145; 25.8%) had at least one documented follow-up encounter. These patients had a median of 1 (25th, 75th percentile: 1, 2) follow-up encounter (range: 1–7 encounters). Among those 145 patients with documented follow-up encounters, the majority (*n* = 103 patients; 71%) had just one follow-up encounter, 18 (12.4%) had two follow-up encounters, and 24 (16.6%) had three or more follow-up encounters. Generally, the 563 initial encounters had more NCPT documented per encounter than the 224 follow-up encounters, with a median of 15 (25th, 75th percentile: 10, 21) terms documented per initial encounter and a median of 12 (25th, 75th percentile: 9, 16) terms per follow-up encounter.

### RDN knowledge of the diabetes EBNPG

The median (25th, 75th percentile) score on the Diabetes EBNPG knowledge test increased from 9 (8, 9) out of 12 points pre-midpoint training to 11 (9, 12) out of 12 points post-training (*p* < 0.001).

### Patient nutrition diagnoses

Across the 787 encounters, 820 nutrition diagnoses were documented using 47 unique NCPT. A comprehensive list of nutrition diagnoses and their frequencies are included in the Supplementary material. The most frequently documented patient diagnoses were “excessive carbohydrate intake” (*n* = 225 patients, 40%), “food and nutrition related knowledge deficit” (*n* = 110 patients, 20%), and “excessive energy intake” (*n* = 77 patients, 14%).

### Alignment with the diabetes EBNPG

Implementation of Diabetes EBNPG recommendations across all 787 encounters and by encounter type (initial or follow-up) is described in Table 2. Across all encounters, 67% had at least one of the 13 recommendations fully (congruence score 4 points) implemented. Almost all encounters (99%) had evidence that at least one of the 13 recommendations was implemented when a lower threshold including both partial and full implementation (congruence score 2–4) was used. Per encounter, the median number of recommendations at least partially implemented was 7 (25th, 75th percentile: 5, 10) and fully implemented was 1 (25th, 75th percentile: 0, 2). Of 4,518 partially implemented recommendations, 2,392 (53%) were “interrupted” and 1,601 (35%) were “deferred.” Per initial encounter, the median number of recommendations at least partially implemented was 8 (25th, 75th percentile: 6, 11) and fully implemented was 1 (25th, 75th percentile: 0, 3). Per follow-up encounter, the median number of recommendations at least partially implemented was 6 (25th, 75th percentile: 4, 8) and fully implemented was 1 (25th, 75th percentile: 0, 2). This corresponds to less frequent implementation of almost all recommendations at follow-up compared to initial encounters.

**Table 2:** Percentage of documented registered dietitian nutritionist (RDN) encounters with patients with diabetes in which each imperative intervention recommendation in the diabetes type 1 and 2 evidence-based nutrition practice guideline was partially or fully implemented as assessed by the diabetes expected care plan analyzer (*n* = 787 patient encounters for 562 patients).

	**Recommendation**	**Statement rating^a^**	**Encounters (*n* = 787) in which recommendation was partially or fully implemented across entire study period^b^ *n* (%)**	**Initial encounters (*n* = 563) in which recommendation was partially or fully implemented *n* (%)**	**Follow-up encounters (*n* = 224) in which recommendation was partially or fully implemented *n* (%)**
1	Individualize nutrition prescription^c^	Fair			
	Theme 1: food and nutrition intake		496 (62.9)	375 (66.6)	121 (54.0)
	Theme 2: diet history		271 (34.4)	194 (34.5)	77 (34.4)
	Theme 3: knowledge, skills, beliefs, and attitudes		137 (17.4)	114 (20.2)	23 (10.3)
	Theme 4: safe food availability		6 (0.8)	4 (0.7)	2 (0.9)
	Theme 5: metabolic balance		389 (49.4)	298 (52.9)	91 (40.6)
2	Individualize macronutrient composition	Fair	671 (85.2)	494 (87.7)	177 (79.0)
3	Encourage fiber intake	Fair	51 (6.5)	43 (7.6)	8 (3.6)
4	Educate about substitution of non-nutritive sweeteners	Fair	502 (63.7)	376 (66.8)	126 (56.3)
5	Advise against excessive intake of nutritive sweeteners	Fair	560 (71.1)	415 (73.7)	145 (64.7)
6	Educate on intake of FDA-approved non-nutritive sweeteners	Weak	294 (37.3)	253 (44.9)	41 (18.3)
7	Educate about substitution of FDA-approved non-nutritive sweeteners	Fair	570 (72.3)	423 (75.1)	147 (65.6)
8	Educate on protein intake and hypoglycemia in adults with diabetes	Fair	392 (49.7)	306 (54.4)	86 (38.4)
9	Encourage cardioprotective eating pattern	Strong	518 (65.7)	399 (70.9)	119 (53.1)
10	Encourage individualized reduction in sodium intake	Fair	239 (30.3)	213 (37.8)	26 (11.6)
11	Encourage individualized physical activity plan	Strong	156 (19.8)	129 (22.9)	27 (12.1)
12	Education on glucose monitoring	Fair	623 (79.1)	463 (82.2)	160 (71.4)
13	Co-ordination of care	Strong	11 (1.4)	7 (1.2)	4 (1.8)

FDA, Food and Drug Administration.

a A strong recommendation rating is supported by good/excellent evidence and guideline author consensus that benefits exceed harm (positive recommendations) or harms exceed benefits (negative recommendation). A fair recommendation rating is supported by lower quality evidence and guideline author consensus that benefits exceed harm (positive recommendations) or harms exceed benefits (negative recommendation). A weak recommendation rating is only supported by low quality evidence or by high quality studies that have not demonstrated that one approach is better than another (24).

b Assessed based on use of natural language processing techniques to automatically count the number of matching nutrition care process terminology (NCPT) between the RDNs' Academy of Nutrition and Dietetics Health Informatic Infrastructure (ANDHII) documentation for a patient encounter and the expected care plans (sets of expected NCPT, should a recommendation be implemented) for the 13 imperative intervention recommendations from the Diabetes evidence-based nutrition practice guideline. A recommendation was considered partially implemented if there were matching NCPT for some domains of the nutrition care process (NCP) and fully implemented if there were matching NCPT for each domain of the NCP.

c The Individualize Nutrition Prescription recommendation statement outlines several specific patient-related factors that could be considered when recommending one eating pattern over another. Five different expected care plans were therefore developed for this recommendation, with each expected care plan including evidence terms that were specific to that patient-related factor.

The recommendation to “individualize macronutrient composition” was partially or fully implemented in 85% of all encounters, followed closely by “education on glucose monitoring,” which was partially or fully implemented in 79% of all encounters. These were the top two most frequently implemented recommendations both in initial and follow-up encounters and also pre- and post-training. The “safe food availability” theme for the recommendation to “individualize nutrition prescription” and the “co-ordination of care” recommendation were not fully implemented in any encounter and were partially implemented in 0.8 and 1.4% of all encounters, respectively. These were the only two recommendations for which there was a small increase in implementation from initial to follow-up encounters (safe food availability: 0.7% during initial encounters to 0.9% for follow-up encounters; co-ordination of care: 1.2–1.8%).

**Figure 1:**
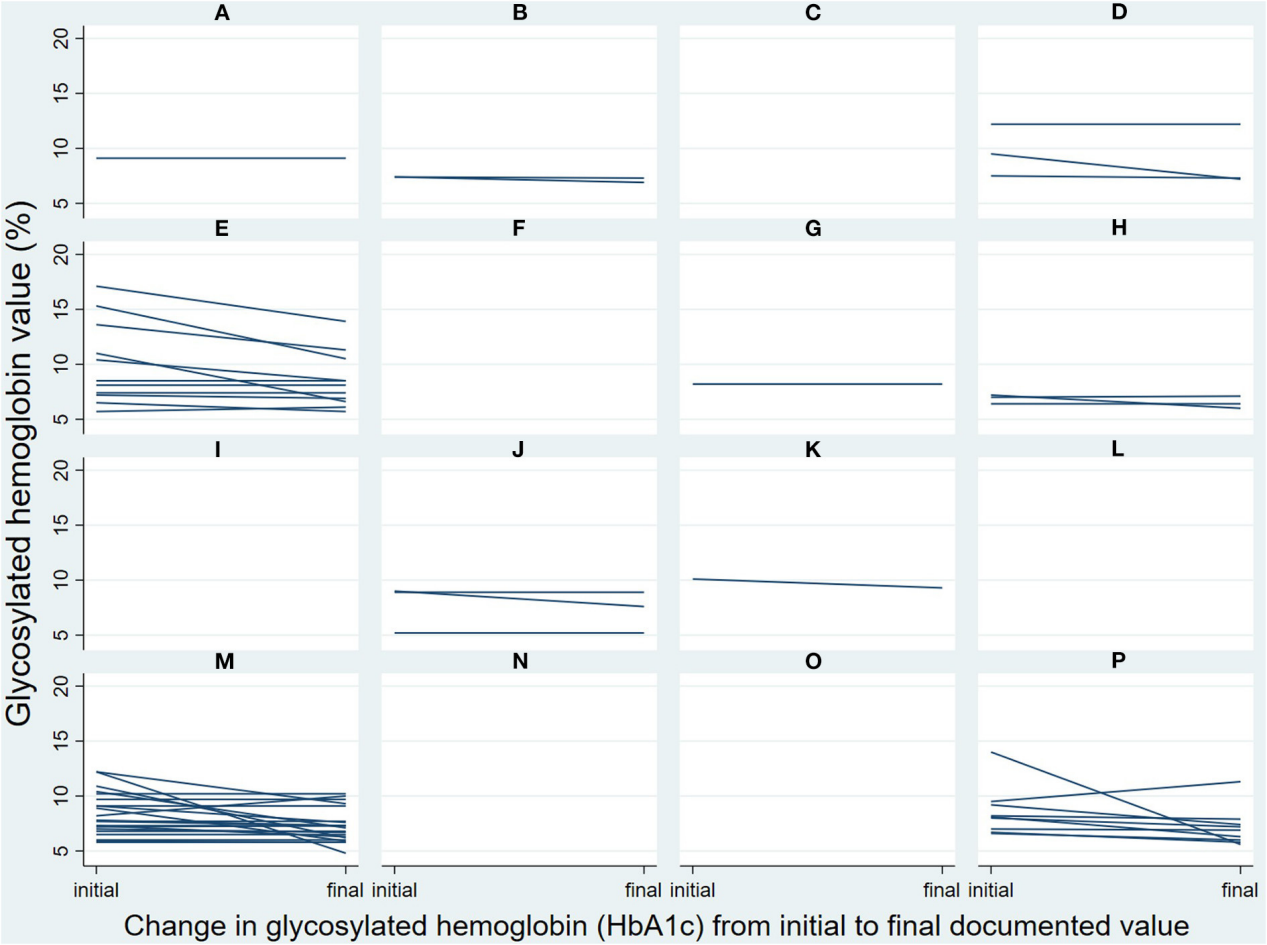
Changes in patients' glycosylated hemoglobin from initial to final documented value, by registered dietitian nutritionists [RDNs] (panels **(A–P)** each represent one RDN) and patient (each individual line) as documented during the Diabetes Registry Study (*n* = 56). Patients had two or more documented RDN encounters, with most having two total encounters (*n* = 36; 64%) and the rest having 3–4 total encounters (*n* = 15; 27%) or 5–8 total encounters (*n* = 5; 9%).

HbA1c, fasting glucose, and body weight were documented during multiple encounters by 10, five, and four RDNs for 56, 20, and 12 patients, respectively. Changes from initial to final documented HbA1c for their individual patients (*n* = 56) are presented in Figure 1 by RDN. There was substantial variation in frequency of documentation and in change in patient HbA1c across RDNs, with three RDNs (Figure 1; panels E, M, P) responsible for the majority of the patients (*n* = 42; 75%) that had multiple documented HbA1c values.

Outcomes that were almost never documented longitudinally included triglycerides (*n* = 3 RDNs, 6 patients), total carbohydrate intake (*n* = 3 RDNs, 5 patients), total cholesterol (*n* = 2 RDNs, 4 patients), and BMI (*n* = 2 RDNs, 3 patients). Blood pressure, medication use and adherence, and quality of life were not longitudinally documented, and patient sociodemographic information was rarely documented.

### Impact of midpoint training

The mean encounter congruence scores (*n* = 519 encounters pre-training; *n* = 204 encounters post-training) did not change from the pre-to-post training documentation period (β = −0.06, SE = 0.04; 95% CI: −0.14, 0.03; *p* = 0.19). The intraclass correlation coefficients for RDN and patient were 0.36 (95% CI: 0.21, 0.54) and 0.78 (0.73, 0.82), respectively.

## Discussion

This study aimed to describe alignment of RDNs' documented nutrition care with the Academy's EBNPG for Type 1 and Type 2 diabetes and examine the impact of a midpoint training on alignment with the guideline. At least one of the 13 imperative recommendations was fully implemented in two-thirds of encounters, and almost all encounters included partial or full implementation of at least one recommendation. However, the midpoint guideline training did not result in the hypothesized improvements in alignment of RDN care with the EBNPG. The most frequently implemented EBNPG recommendations were related to improving glycemic control. RDNs infrequently documented initial and follow-up values for the major outcome measures highlighted in the Diabetes EBNPG.

Documentation for two-thirds of encounters was consistent with full delivery of at least one imperative recommendation from the Diabetes EBNPG. This aligns with findings of an observational study of 61 general practitioners in the Netherlands (25), which found that 47 clinical practice guideline recommendations were followed in 61% of 12,880 practitioner self-reported decisions. The study authors noted that recommendations that are consistent with practice norms are more likely to be implemented; recommendations they deemed controversial (not consistent with prevailing norms in practice) and/or vague were only implemented about one-third of the time, compared to about two-thirds of the time for non-controversial and/or clear recommendations (25).

In our study, the guideline developers rated 12 of the 13 recommendations as fair or strong. Strong or fair ratings mean that the recommendations are supported by evidence and guideline developer consensus regarding the risk/benefit profile and are more likely to be consistent with prevailing norms in practice. There was documented evidence of implementation for many of the recommendations with fair ratings (e.g., individualize macronutrient composition, education on glucose monitoring, advise against excessive intake of nutritive sweeteners). However, some recommendations with a strong rating (e.g., encourage individualized physical activity plan, co-ordination of care) were infrequently implemented. This is perhaps not surprising, as most patients (~75%) only had one documented RDN encounter over a 1-year time frame. This is consistent with studies that found only about 1 in 10 people with diabetes attended at least one RDN or other diabetes education visit or class (26, 27). In this study, RDN documented care was focused heavily on improving glycemic management, which is generally considered fundamental for diabetes care. Most patients (71%) that had documented follow-up encounters had only one additional RDN encounter. With a limited number of visits, there may not have been time to address important aspects of holistic diabetes care, such as providing comprehensive preventive guidance and treatment for common comorbidities of diabetes (e.g., cardiovascular disease and chronic kidney disease) and engaging in co-ordination of care to ameliorate social determinants of health (e.g., food insecurity). There was a very small increase in implementation of recommendations related to safe food availability and co-ordination of care in follow-up compared to initial encounters, although with 93% of patients receiving one or two encounters it is not surprising that the focus remained on glycemic management in follow-up encounters. These findings identify opportunities to improve the current infrastructure to support implementation of the diabetes guideline and more comprehensive diabetes education *via* additional RDN encounters for patients. Approaches that could be considered include increasing patient and medical provider awareness of current insurance coverage for MNT, expanding MNT insurance coverage, and addressing other barriers, such as lack of patient transportation or time *via* telehealth sessions or RDN co-location in medical practices (28–30).

Given the registry design of the study, documentation of the outcome measures highlighted in the Diabetes EBNPG could not be standardized or mandated. RDNs in this study needed to document into ANDHII in addition to documenting into their electronic medical record, and may have not documented these values into ANDHII because they are already routinely and separately captured in the patient medical record. In the future, training and job aids could address the importance of capturing these outcomes as part of the detailed documentation of standard RDN care into ANDHII, to allow for evaluation of the impact of care that is consistent with EBNPG on patient outcomes.

Contrary to our hypothesis, the midpoint training had little effect on alignment of RDN care with the guideline. This is consistent with a pilot study showing only a small improvement (4%) in the alignment between RDN documented care and the Diabetes Prevention EBNPG after guideline training (10). It is likely that the midpoint training intervention was not intensive enough to strongly impact guideline implementation. In an examination of 41 studies with quantitative assessment of implementation of mental health guidelines, Bauer noted that interventions that were successful in improving guideline implementation were resource intensive and tended to involve system redesign (31). Similarly, a systematic meta-review of studies examining factors that influence implementation of clinical guidelines for healthcare professionals also emphasized that most reviews found that effective implementation strategies had multiple components (11).

### Limitations

A major strength of this study is the collection and analysis of real-world data on typical diabetes care documented by RDNs in outpatient settings. This study also has limitations. Fundamentally, documented care may not fully reflect the care that was provided to patients. We cannot rule out that some details of patient initial encounters and follow-up encounters were not recorded in ANDHII, meaning that we may have underestimated recommendation implementation. In particular, the documentation for follow-up care contained fewer terms per encounter, compared to documentation of initial care, which may have resulted in greater underestimation of recommendation implementation in follow-up compared with initial encounters. It also might be expected, however, that follow-up visits are shorter than initial visits, and as a result, fewer recommendations would be implemented per follow-up encounter. It is also possible that we underestimated actual patient contact with the RDN. However, our findings regarding patient contact with the RDN are consistent with established underutilization of diabetes education programs and MNT (26, 27, 32). We did not capture patient interaction with other forms of diabetes support, such as group diabetes management classes. We may have overestimated recommendation implementation by using an automated analyzer (18) that may not distinguish nuances of care and a relatively low threshold to assess congruence with the EBNPG that included partial implementation. Patient measures highlighted in the EBNPG were only documented for a small subset of the patients and RDNs, preventing us from doing more than describing frequency of documentation and visually depicting changes in HbA1c. We did not have information on diabetes medication use, which can also result in changes in HbA1c. In future studies, efforts should be made to improve the consistency with which information on patient measures and medication use is documented to best estimate the impact of medical nutrition therapy on HbA1c. Finally, a small convenience sample of RDNs from a few parts of the United States participated, and there was high RDN dropout, limiting the generalizability of the results and the ability to detect subtle changes in guideline implementation as a result of the training.

## Conclusion

In this real-world dataset on the outpatient MNT provided to adult patients with diabetes, most RDN encounters had documented evidence that at least one recommendation from the EBNPG was implemented. The most frequently implemented EBNPG recommendations were related to improving glycemic control. A midpoint training focused on EBNPG content had no impact on alignment between RDN's documented care and the EBNPG.

## Data availability statement

The raw data supporting the conclusions of this article will be made available by the authors, without undue reservation.

## Ethics statement

The studies involving human participants were reviewed and approved by the American Academy of Family Physicians (AAFP) Institutional Review Board (IRB). The AAFP IRB determined the project was not research involving human subjects based on Office for Human Research Protections Guidance on Research Involving Coded Private Information or Specimens (#17-287). Written informed consent for participation was not required for this study in accordance with the national legislation and the institutional requirements.

## Author contributions

EL-J and EJ oversaw study implementation and data acquisition. EL-J, KKe, CP, DS, JP, and EJ conducted data analysis and all authors contributed to data interpretation. EL-J and KKe wrote the first draft of the manuscript. All authors reviewed and commented on subsequent drafts of the manuscript.

## Funding

Funding for this study was provided by the Diabetes Dietetic Practice Group. The Academy of Nutrition and Dietetics and the Commission on Dietetic Registration provided financial and material support for the development of the Academy of Nutrition and Dietetics Health Informatics Infrastructure.

## Conflict of interest

Authors EL-J, KKe, and CP are employees of the Academy of Nutrition and Dietetics, which has a financial interest in the Academy of Nutrition and Dietetics Health Informatics Infrastructure platform and the Nutrition Care Process Terminology described in this article. Authors EJ and DS have contracts with the Academy of Nutrition and Dietetics. Author KKn received the Diabetes Dietetic Practice Group Karen Goldstein Memorial Grant from the Academy of Nutrition and Dietetics Foundation. The remaining authors declare that the research was conducted in the absence of any commercial or financial relationships that could be construed as a potential conflict of interest.

## Publisher's note

All claims expressed in this article are solely those of the authors and do not necessarily represent those of their affiliated organizations, or those of the publisher, the editors and the reviewers. Any product that may be evaluated in this article, or claim that may be made by its manufacturer, is not guaranteed or endorsed by the publisher.
