# Supplementary material for: A quasi-experimental study provides evidence that registered dietitian nutritionist care is aligned with the Academy of Nutrition and Dietetics evidence-based nutrition practice guidelines for type 1 and 2 diabetes
Source: Front Nutr. 2022 Sep 12;9:969360. doi: 10.3389/fnut.2022.969360 (PMC9511164; doi:10.3389/fnut.2022.969360)
Supplement: Supplementary file 1 [file Table_1.DOCX]

**Supplementary Material**

**Diabetes Registry Study timeline schematic**


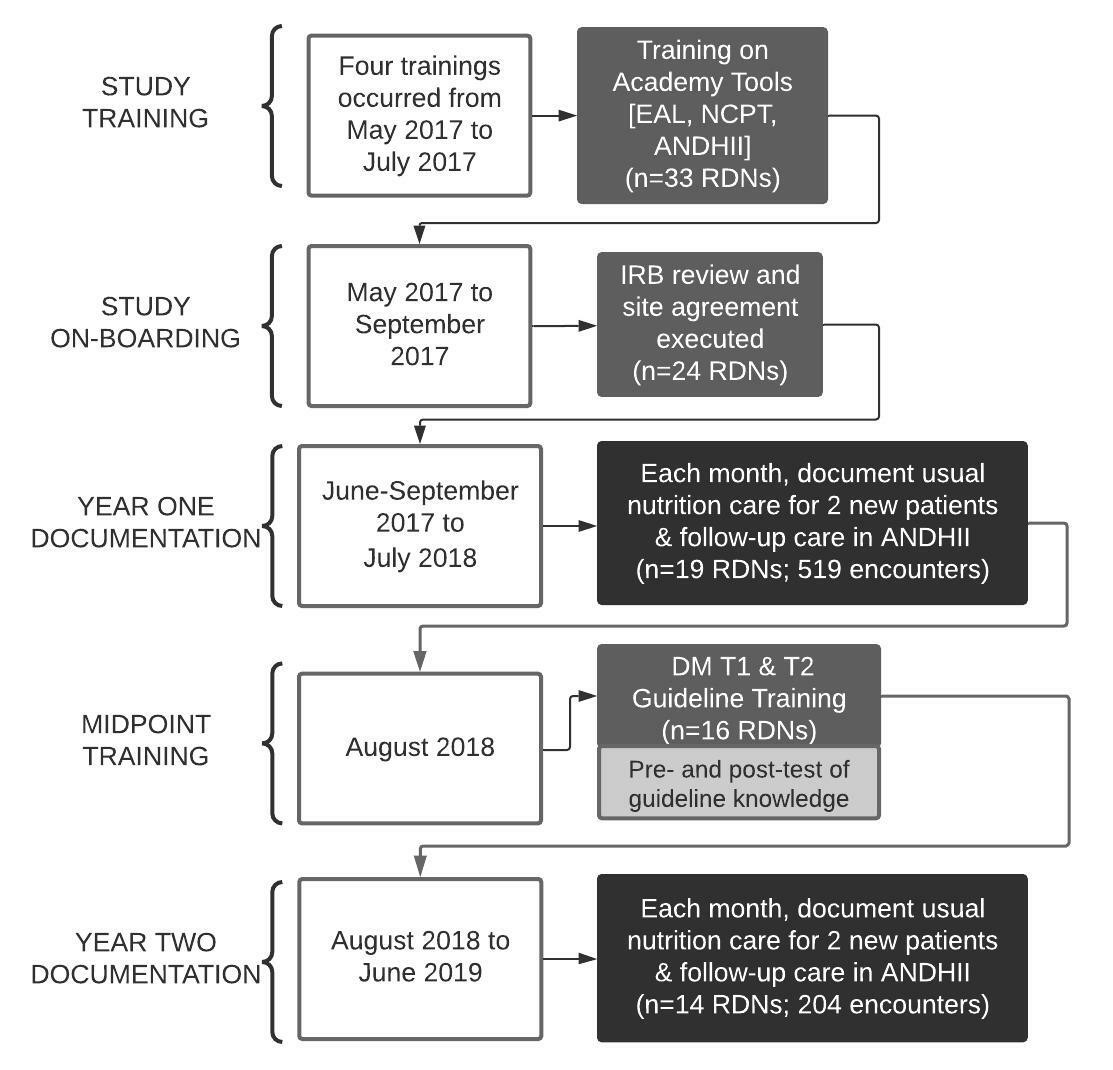


EAL = Evidence Analysis Library, NCPT = Nutrition Care Process Terminology, ANDHII = Academy of Nutrition and Dietetics Health Informatics Infrastructure, IRB = Institutional Review Board, DM T1 & T2 = diabetes mellitus type 1 and type 2, RDN = Registered Dietitian Nutritionist

**Complete list of nutrition care process terminology (NCPT) nutrition diagnoses among adult patients with type 1 and type 2 diabetes documented by registered dietitian nutritionists (RDNs) in the Academy of Nutrition and Dietetics Health Informatics Infrastructure (ANDHII) throughout the Diabetes Registry Study**

| **NCPT Nutrition Diagnosis** | n=562 patients | |
| --- | --- | --- |
|  | n | % |
| Excessive carbohydrate intake | 225 | 40.0 |
| Food and nutrition related knowledge deficit | 110 | 19.6 |
| Excessive energy intake | 77 | 13.7 |
| Altered nutrition related laboratory values | 61 | 10.9 |
| Inconsistent carbohydrate intake | 42 | 7.5 |
| Overweight/obesity^a^ | 41 | 7.3 |
| Physical inactivity | 39 | 6.9 |
| Intake of types of carbohydrate inconsistent with needs | 34 | 6.0 |
| Undesirable food choices | 26 | 4.6 |
| Excessive fat intake | 18 | 3.2 |
| Excessive oral intake | 13 | 2.3 |
| Inadequate fiber intake | 12 | 2.1 |
| Obese, class III^a^ | 10 | 1.8 |
| Inadequate energy intake | 8 | 1.4 |
| Obese, class I^a^ | 7 | 1.2 |
| Excessive sodium intake | 7 | 1.2 |
| Inadequate carbohydrate intake | 7 | 1.2 |
| Obese, class II^a^ | 7 | 1.2 |
| Overweight, adult or pediatric^a^ | 6 | 1.1 |
| Inadequate oral intake | 6 | 1.1 |
| Predicted excessive energy intake | 6 | 1.1 |
| Excessive fluid intake | 5 | 0.9 |
| Not ready for diet/lifestyle change | 4 | 0.7 |
| Excessive protein intake | 4 | 0.7 |
| Inadequate fluid intake | 4 | 0.7 |
| Imbalance of nutrients | 4 | 0.7 |
| Disordered eating pattern | 3 | 0.5 |
| Food medication interaction | 3 | 0.5 |
| Limited adherence to nutrition related recommendations | 3 | 0.5 |
| Predicted suboptimal energy intake | 2 | 0.4 |
| Unintended weight loss | 2 | 0.4 |
| Intake of types of fats inconsistent with needs (specify) | 2 | 0.4 |
| Inadequate calcium intake | 2 | 0.4 |
| Predicted food-medication interaction | 2 | 0.4 |
| Inadequate protein intake | 1 | 0.2 |
| Inadequate protein-energy intake | 1 | 0.2 |
| Underweight | 1 | 0.2 |
| Impaired nutrient utilization | 1 | 0.2 |
| Predicted inadequate nutrient intake (specify) | 1 | 0.2 |
| Biting/chewing (masticatory) difficulty | 1 | 0.2 |
| Enteral nutrition composition inconsistent with needs | 1 | 0.2 |
| Unsupported beliefs/attitudes about food or nutrition related topics | 1 | 0.2 |
| Suboptimal bioactive substance intake | 1 | 0.2 |
| Limited food acceptance | 1 | 0.2 |
| Limited access to food | 1 | 0.2 |
| Excessive alcohol intake | 1 | 0.2 |
| No nutrition diagnosis at this time | 1 | 0.2 |

^a^ "Overweight/obesity," "overweight, adult or pediatric," and "obese, class I-III" are unique terms that can all be selected from the NCPT.
